# ADHD Dimension, Childhood trauma and Perceived Stress: an observational study on peripartum women affected by mood and anxiety disorders

**DOI:** 10.1192/j.eurpsy.2023.1355

**Published:** 2023-07-19

**Authors:** I. Bilotta, G. Anibaldi, L. Stampatore, C. Concolato, M. Medugno, A. Fattorini, L. Cutillo, S. Bernardi, G. Culicchia, A. Bassi de Toni, A. Del Casale, M. Pompili, G. Angeletti

**Affiliations:** 1 Psychiatry Residency Training Program, Faculty of Medicine and Psychology, Sapienza University of Rome; 2 Department of Neurosciences, Mental Health and Sensory Organs, Faculty of Medicine and Psychology, Sant’Andrea Hospital, Sapienza University of Rome, Rome, Italy

## Abstract

**Introduction:**

Pregnancy is an important life event, involving body and mind transformation as well as brain reorganization. ADHD dimension is an additional aggravating factor, albeit poorly studied in the literature, in patients with mental health disorders in the peripartum.

**Objectives:**

The purpose of this study was to evaluate the correlation among ADHD dimension, trauma in childhood, and anxiety-depressive symptomatology to assess whether the ADHD dimension may affect the quality of life of peripartum patients, and to identify vulnerability factors and self-harm risk.

**Methods:**

The sample included 74 women aged 21-46 years, recruited from Sant’Andrea Hospital in Rome between 2015 and 2019. All recruited women were administered the following scales: Adult ADHD Self Report Scale (ASRS), Edinburgh Postnatal Depression Scale (EPDS); Childhood Trauma Questionnaire (CTQ), Perceived Stress Scale (PSS); Minnesota Multiphasic Personality Inventory (MMPI). Statistical analysis was performed by Pearson’s correlation with SPSS software to verify the presence of linear relationships (p<0.05) among theADHD dimension, assessed by the ASRS scale, and the other psychopathological dimensions.

**Results:**

The sample was divided into two groups depending on the results of ASRS: 26 patients were positive for at least one of the ASRS scale items, while 48 patients were negative. The groups did not statistically differ in socio-demographic variables examined. The medium score at EPDS was 15,11 (± 8,43). It was found that the severity of ADHD dimension directly correlated with the duration of mental symptoms in peripartum (r=0.324;p=0.013), with the total CTQ scale score (r=0.342; p=0.004), with emotional abuse detected by CTQ (r=0.415; p<0.001), with emotional neglect detected by CTQ (r=0.291; p=0.014) and with perceived stress detected by PSS scale (r=0.456; p<0.001). Furthermore, we identified a correlation between self-injurious ideation and ADHD symptomatology (r=0.269; p =0.049) evaluating the item 10 of EPDS.

**Conclusions:**

The severity of ADHD traits directly correlates with the symptomatology and duration of mental disorder in peripartum. Specifically, ADHD patients who develop anxious-depressive symptoms are more likely to have experienced emotional abuse and emotional neglect in childhood.

**Disclosure of Interest:**

None Declared

